# Antibacterial Activity and Mechanism of Canagliflozin against Methicillin-Resistant *Staphylococcus aureus*

**DOI:** 10.3390/molecules28155668

**Published:** 2023-07-26

**Authors:** Siyao Gu, Bing Fan, Fang Wan, Tong Gao, Yuanyuan Qi, Jin Zhou, Yaou Zhang, Dayong Gu, Weidong Xie

**Affiliations:** 1 State Key Laboratory of Chemical Oncogenomics, Shenzhen International Graduate School, Tsinghua University, Shenzhen 518055, China; gusy22@mails.tsinghua.edu.cn (S.G.); wanf21@mails.tsinghua.edu.cn (F.W.); gao-t21@mails.tsinghua.edu.cn (T.G.); qyy21@mails.tsinghua.edu.cn (Y.Q.); zhangyo@sz.tsinghua.edu.cn (Y.Z.); 2 Open FIESTA Center, Shenzhen International Graduate School, Tsinghua University, Shenzhen 518055, China; 3 Shenzhen Key Laboratory of Health Science and Technology, Institute of Biopharmaceutical and Health Engineering, Shenzhen International Graduate School, Tsinghua University, Shenzhen 518055, China; 4 Department of Laboratory Medicine, Shenzhen Institute of Translational Medicine, The First Affiliated Hospital of Shenzhen University, Shenzhen Second People’s Hospital, Shenzhen 518035, China; qixin20122016@163.com; 5 Institute for Ocean Engineering, Shenzhen International Graduate School, Tsinghua University, Shenzhen 518055, China; zhou.jin@sz.tsinghua.edu.cn

**Keywords:** canagliflozin, diabetic foot infections, methicillin-resistant *Staphylococcus aureus*, bacterial infection

## Abstract

Diabetic foot infection (DFI) is a common complication in diabetes patients, with foot infections being the leading cause of amputations. *Staphylococcus aureus* is frequently found in diabetic foot infections, of which methicillin-resistant *Staphylococcus aureus* (MRSA) has become a major clinical and epidemiological challenge. Since MRSA strains are resistant to most β-lactam antibiotics, and also partially resistant to other antibiotics, treatment is difficult and costly. The emergence of drug-resistant bacteria often arises from overuse or misuse of antibiotics. Clinically, canagliflozin is commonly used for the treatment of type 2 diabetes. On this basis, we investigated the antibacterial activity and mechanism of canagliflozin against MRSA, with the aim to discover novel functions of canagliflozin and provide new insights for the treatment of MRSA. Using the microbroth dilution method to determine the half maximal inhibitory concentration of drugs, we found that canagliflozin not only can inhibit the growth of methicillin-sensitive *Staphylococcus aureus* (MSSA) but also exhibits antibacterial activity against MRSA. The IC50 values, at approximately 56.01 μM and 57.60 μM, were almost the same. At 12 h, canagliflozin showed a significant antibacterial effect against MRSA at and above 30 μM. In addition, its combined use with penicillin achieved better antibacterial effects, which were increased by about three times. Additive antibacterial activity (FICI = 0.69) was found between penicillin and canagliflozin, which was better than that of doxycycline and canagliflozin (FICI = 0.95). Canagliflozin also affected bacterial metabolic markers, such as glucose, ATP, and lactic acid. The results of crystal violet staining indicate that canagliflozin disrupted the formation of bacterial biofilm. Our electron microscopy results showed that canagliflozin distorted the bacterial cell wall. The results of RT-PCR suggest that canagliflozin down-regulated the expressions of biofilm-related gene (clfA, cna, agrC, mgrA, hld) and methicillin-resistance gene (mecA), which was related to MRSA. Molecular docking also indicated that canagliflozin affected some interesting targets of MRSA, such as the sarA, crtM and fnbA proteins. In conclusion, canagliflozin exhibits antibacterial activity against MRSA by affecting bacterial metabolism, inhibiting its biofilm formation, distorting the bacterial cell wall, and altering the gene expression of biofilm formation and its virulence. Our study reveals the antibacterial activity of canagliflozin against MRSA, providing a new reference for treating diabetic foot infections.

## 1. Introduction

Diabetic foot is a common complication in diabetic patients [1] and is caused by diabetic neuropathy and circulatory problems. This can lead to an increased susceptibility to foot infections, resulting in ulcers and ultimately limb amputations [2]. This condition is common among diabetic patients worldwide, with studies showing that the prevalence of foot ulcers in diabetic patients ranges from 1.5% to 10% [3,4]. Over 5% of diabetic patients have a medical history of foot ulcers, and there are about 18.6 million cases of diabetic foot infection (DFI) worldwide each year [5,6]. The common bacteria in respect of DFI include *Staphylococcus aureus*, *streptococci*, and *Escherichia coli* [6,7]. *Staphylococcus aureus* is one of the most common pathogens [8], and diabetic patients are 2.8 times more likely to be infected by the bacteria compared to healthy people due to elevated blood glucose levels. Bacterial infection can lead to compromised immune function, thereby enhancing the propagation and growth of bacterial or fungal pathogens [9].

Penicillin, tetracycline, and other antibiotics are common clinical treatment options for *Staphylococcus aureus* [10]. Penicillin was the first effective antibiotic capable of treating human diseases due to its high yield and low prices while being a potent drug [11]. Thus, it is still the drug of choice for sensitive bacteria. However, this drug has a narrow antibacterial spectrum and is unstable against the β-lactamase produced by bacteria, leading to the development of drug-resistant bacteria [11]. Due to the disadvantages of penicillin, tetracyclines have been a focus of antibiotic research. In recent years, two tetracycline derivatives have been released on the market; namely, elacycline and omacycline. These derivatives have broad-spectrum antibacterial activity, which can enhance bactericidal activity and reduce side effects in patients [12]. Tetracycline is a relatively new type of mature antibacterial drug, and it has been applied in clinical practice. Doxycycline and minocycline are also commonly used in clinical treatment but still have certain risks in respect of antibiotic resistance and toxic side effects.

Due to the overuse of these antibiotics, drug-resistant bacteria such as methicillin-resistant *Staphylococcus aureus* (MRSA) have emerged [13]. MRSA is not only resistant to methicillin but also to all other β-lactam antibiotics. It also exhibits varying degrees of resistance to aminoglycosides, macrolides, tetracyclines, fluoroquinolones, sulfonamides, and rifampin, though it remains sensitive to vancomycin [14]. MRSA can lead to a variety of serious infections such as pneumonia, sepsis, and endocarditis, as well as causing a mortality rate 64% higher than that of infections by sensitive bacteria [15]. The broad-spectrum resistance of MRSA not only makes treatment difficult, but also creates an increase in potential complications. Therefore, it makes sense to find effective drugs to treat MRSA infections.

Canagliflozin is a hypoglycemic drug used to treat type 2 diabetes. As an inhibitor of sodium–glucose cotransporter 2 (SGLT2), it reduces blood glucose levels by inhibiting SGLT-2, thereby decreasing renal reabsorption of filtered glucose [16]. Canagliflozin promotes urinary glucose excretion, increases energy expenditure, aids in weight loss, and reduces cardiovascular risk [17,18]. Moreover, canagliflozin has anti-inflammatory [18,19,20,21,22], anti-aging [23] and anti-tumor effects [24]. It is also proposed for use in the treatment of autoimmune diseases [25] and improves the gut microbiota [26,27]. Our study further explores the functionality of canagliflozin, investigating its antibacterial activity and mechanisms against MRSA.

## 2. Results

### 2.1. Antibacterial Activity of Canagliflozin, Penicillin and Doxycycline and Their IC50 Values

**Figure 1:**
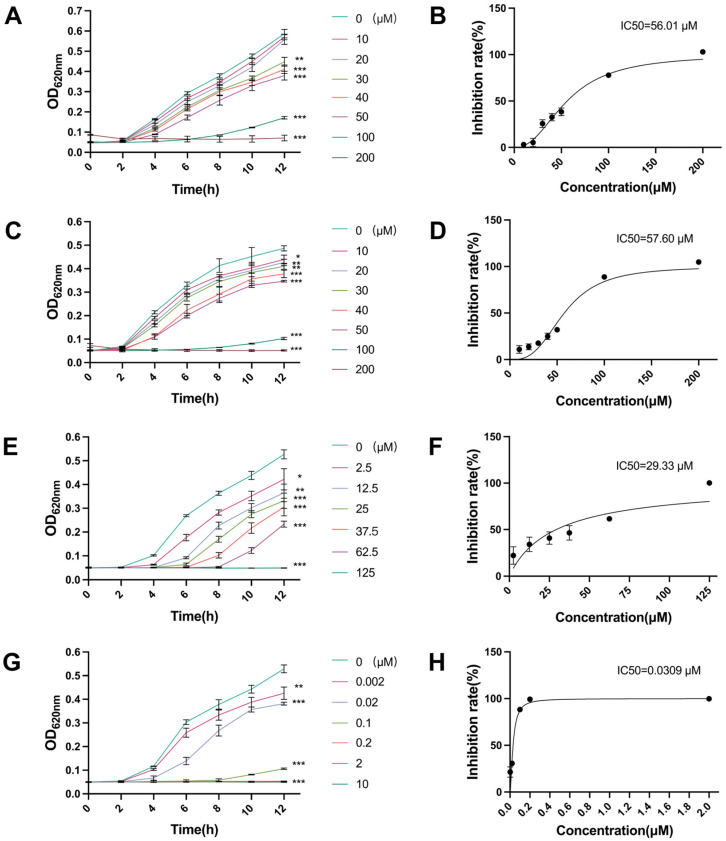
Antibacterial effects of CAN, PNC, and DOX and their IC50 values. (**A**) MRSA growth curves at different concentrations of CAN. (**B**) The IC50 value of CAN against MRSA at the 12th hour. (**C**) MSSA growth curves at different concentrations of CAN. (**D**) The IC50 value of CAN against MSSA at the 12th hour. (**E**) MRSA growth curves at different concentrations of PNC. (**F**) The IC50 value of PNC against MRSA at the 12th hour. (**G**) MRSA growth curves at different concentrations of DOX. (**H**) The IC50 value of DOX against MRSA at the 12th hour. * *p* < 0.05, ** *p* < 0.01, *** *p* < 0.001 vs. control (concentration = 0 µM).

First, we evaluated the antibacterial activity of canagliflozin (CAN), penicillin (PNC), and doxycycline (DOX) and determined their IC50 values. PNC and DOX as classic antibiotics were used to compare the antibacterial effect of CAN. At 12 h, the antibacterial effect of CAN against MRSA at 30 μM appeared significant (Figure 1A), PNC showed a significant antibacterial effect at 2.5 μM or above, while DOX had significant antibacterial effect at a concentration of 0.002 μM (Figure 1E,G). The IC50 values of CAN, PNC, and DOX against MRSA were 56.01 μM, 29.33 μM, and 0.0309 μM, respectively. Since MRSA is mainly resistant to PNC, the antibacterial effect of PNC was actually poorer than DOX. We also used these drugs on MSSA to explore the specificity of their antibacterial effects (Figure 1C and Figure S1). The IC50 value of CAN against MSSA was 57.60 μM, while PNC and DOX could completely inhibit the growth of MSSA at concentrations below 2.5 μM and 0.1 μM, respectively (Figure S1). The results indicated that CAN had the same antibacterial activity against MSSA as against MRSA. Its antibacterial activity was not affected by the antibiotic resistance of the bacteria.

### 2.2. Antibacterial Effect of CAN Combined with PNC and DOX

**Figure 2:**
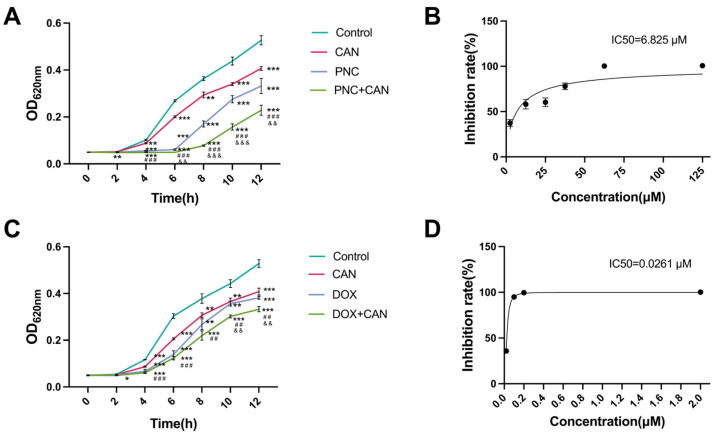
Effects of CAN, PNC, DOX, PNC + CAN, and DOX + CAN on MRSA growth. (**A**) Antibacterial effect of CAN combined with PNC. (**B**) The IC50 value of PNC after combination of PNC and CAN. (**C**) Antibacterial effect of CAN combined with DOX. (**D**) The IC50 value of DOX after combination of DOX and CAN. * *p* < 0.05, ** *p* < 0.01, *** *p* < 0.001 vs. control; ^##^ *p* < 0.01, ^###^ *p* < 0.001 vs. CAN; ^&&^ *p* < 0.01, ^&&&^ *p* < 0.001 vs. PNC or DOX.

When 25 μM PNC was used in combination with 50 μM CAN, the combined effect of the two drugs displayed significant differences compared with the single PNC after 6 h (Figure 2A). At 12 h, the IC50 value of PNC decreased to 6.825 μM after the combination (Figure 2B). The change in the IC50 value suggested that the antibacterial effect was increased by about three times. Strong additive antibacterial activity (FICI = 0.69) was found between PNC and CAN (MIC_PNC(combination)_ = 42.5 μM, MIC_PNC_ = 125 μM, MIC_CAN(combination)_ = 70 μM, MIC_CAN_ = 200 μM). When 0.02 μM DOX was used in combination with 50 μM CAN, the results showing significant differences from the effect of only DOX manifested 10 h later (Figure 2C). At 12 h, the IC50 value of DOX after combination was 0.0261 μM (Figure 2D). Weak additivity (FICI = 0.95) was also observed between DOX and CAN (MIC_DOX(combination)_ = 0.1 μM, MIC_DOX_ = 0.2 μM, MIC_CAN(combination)_ = 90 μM, MIC_CAN_ = 200 μM). It can be seen that the combination of PNC and CAN had a better antibacterial effect, not only reflected in the improvement of IC50 value, but also in the FICI.

### 2.3. CAN Inhibits Bacterial Biofilm and Metabolism

**Figure 3:**
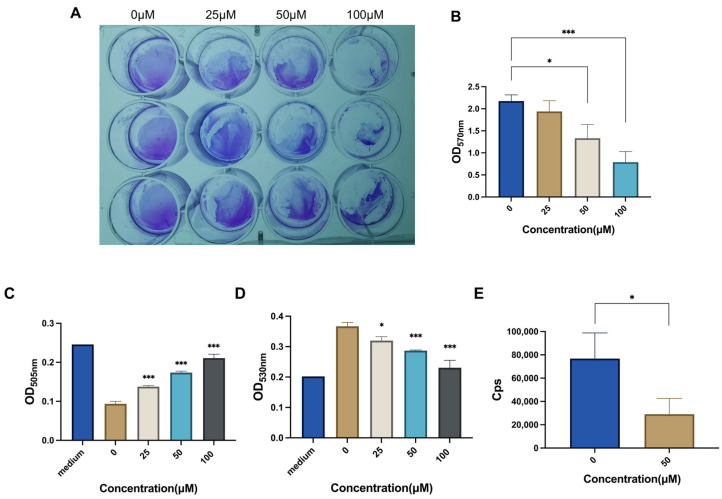
Effects of CAN on MRSA biofilm formation and metabolism. (**A**) Biofilm formation of MRSA under different CAN concentrations. (**B**) Comparison of biofilm growth. (**C**) Comparison of glucose surplus in medium. (**D**) Comparison of lactic acid in medium. (**E**) Comparison of ATP content in bacterial protein. * *p* < 0.05, *** *p* < 0.001 vs. Control (concentration = 0 μM).

The formation of biofilms plays an important role in the development of *Staphylococcus aureus* and its drug resistance [28,29], so we explored the antibacterial mechanism of CAN in this respect. The results of crystal violet staining indicate that CAN significantly inhibited the growth of MRSA biofilms, with increasing effectiveness as the concentration increased (Figure 3A,B). Therefore, it is suggested that CAN may inhibit bacterial growth by disrupting bacterial biofilms.

We further explored the effects of CAN on bacterial metabolism. The ATP test results suggest that the energy of bacteria in the CAN-treated group was significantly lower than that in the control group (Figure 3E). Additionally, due to the rapid growth of MRSA, the glucose within the nutrient medium was exhausted at 12 h. Therefore, we decided to test the glucose and lactic acid in the nutrient medium after 4 h of culture instead of the originally planned 12 h. The results show that the residual glucose in the nutrient medium rose with the increase in drug concentration (Figure 3C). Within 4 h, the bacteria had produced lactic acid. However, the lactic acid gradually decreased as the concentration of CAN increased, thus indicating the inhibition of bacterial respiration due to the drug (Figure 3D). In conclusion, CAN may affect bacterial metabolism. 

In addition, the results of SDS-PAGE showed that CAN may not affect the total soluble protein inside MRSA (Figure S2).

### 2.4. Field-Emission Scanning Electron Microscopy (FESEM)

**Figure 4:**
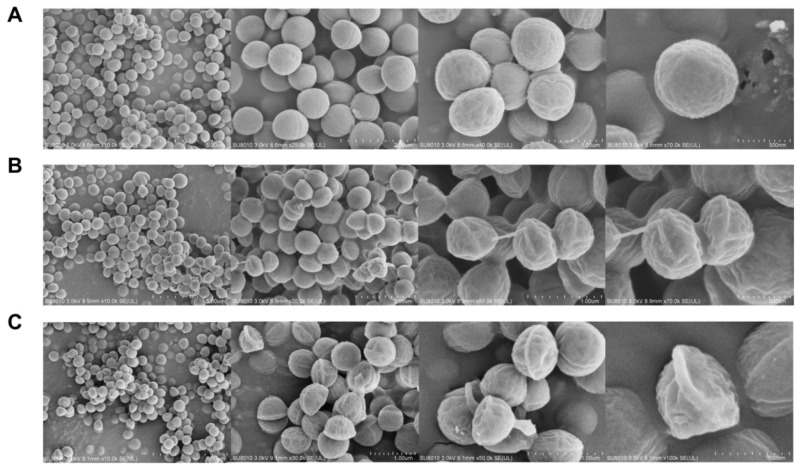
Field-emission scanning electron micrographs of MRSA. (**A**) Untreated control MRSA cells at 12 h post-inoculation. (**B**) MRSA cells treated with CAN at 50 μM for 12 h. (**C**) MRSA cells treated with CAN at 100 μM for 12 h.

FESEM was used to observe the effects of CAN on bacterial morphology. The untreated MRSA cells exhibited smooth surfaces without folds and gullies once fully grown. However, after being treated with CAN, the cell wall of MRSA became distorted, resulting in cellular damage and wrinkles. Under different magnifications, it was shown that the growth state of the bacteria after CAN treatment was poor. With the CAN concentration increased, the number of cells became lower, and the shapes of the cells were more irregular and shrunken (Figure 4). The results of FESEM directly reflected the impact of CAN on the cell wall and the morphology of MRSA.

### 2.5. CAN Affects Biofilm-Related Signaling Pathways in MRSA

**Figure 5:**
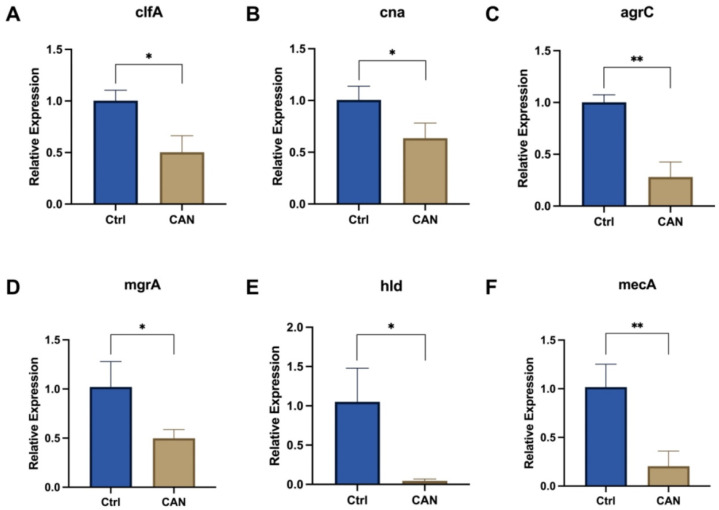
CAN altered the expression of genes involved in the MRSA biofilm signaling pathway. (**A**) The regulatory effect of CAN on clfA. (**B**) The regulatory effect of CAN on cna. (**C**) The regulatory effect of CAN on agrC. (**D**) The regulatory effect of CAN on mgrA. (**E**) The regulatory effect of CAN on hld. (**F**) The regulatory effect of CAN on mecA. * *p* < 0.05, ** *p* < 0.01 vs. control.

We investigated the key signaling pathways involved in biofilm formation to better understand the mechanism by which CAN inhibit the formation of MRSA biofilms. In the early stage of biofilm formation, clfA and cna genes related to microbial surface components recognizing adhesive matrix molecules (MSCRAMMs) play an important role [28,30,31,32]. The addition of CAN led to the downregulation of these genes (Figure 5A,B). At the same time, the expression of agrC and mgrA was also downregulated (Figure 5C,D). These are bacterial virulence-related regulatory genes, and mgrA is also a cell-wall-related gene. In addition, the expression of the hld virulence gene, which is regulated by agrC, was also downregulated (Figure 5E). The expression of the mecA gene related to penicillin resistance was downregulated, suggesting that CAN might affect the degree of MRSA drug resistance (Figure 5F). These results show that CAN might affect signaling pathways related to biofilm formation in MRSA.

### 2.6. Analysis of Interaction between CAN and Bacterial Protein

**Figure 6:**
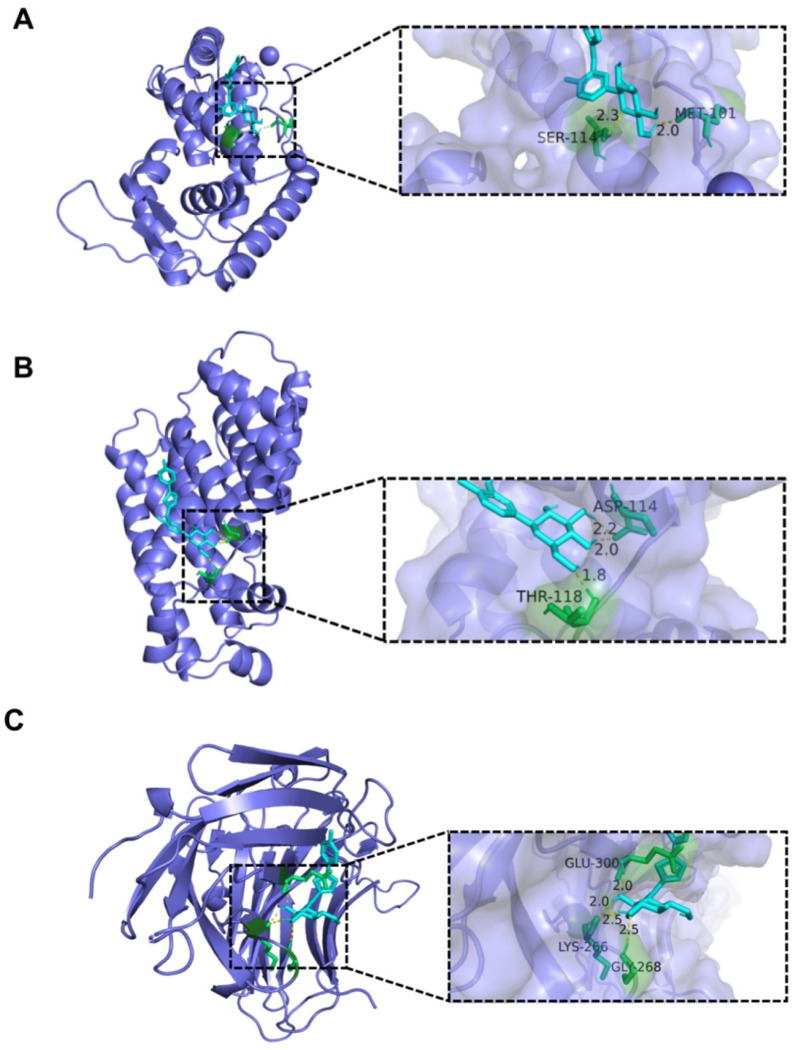
Molecular docking between CAN and bacterial protein active sites. (**A**) The binding poses and interactions of CAN with sarA. (**B**) The binding poses and interactions of CAN with crtM. (**C**) The binding poses and interactions of CAN with fnbA.

Molecular docking analysis was performed to ascertain the affinity of the candidate drugs for their intended targets. For this purpose, we docked CAN to seven proteins (sarA, PDB ID: 2FRH, resolution 2.50 Å; crtM, PDB ID: 2ZY1, resolution 1.78 Å; fnbA, PDB ID: 4B5Z, resolution 2.2 Å; cna, PDB ID: 2F68, resolution 1.95 Å; agrC, PDB ID: 4BXI, resolution 2.2 Å; agrA, PDB ID: 4XYO, resolution 2.0 Å; PBP2a, PDB ID: 5M18, resolution 1.98 Å). PBP2a is a protein expressed by the gene, mecA [30]. The binding poses and energy of CAN with these proteins were obtained with AutoDockTools-1.5.7. CAN was bound to these protein targets through visible hydrogen bonds and strong electrostatic interactions (Figure 6). The results show that sarA had a low binding energy of −7.97 kcal/mol, indicating highly stable binding. In addition, for CAN, crtM and fnbA had low binding energies of −6.5 and −6.03 kcal/mol (binding energy below −5 kcal/mol indicates stable binding). The docking results of these three protein active sites and CAN are shown in Figure 6, while other proteins are shown in Figure S3.

## 3. Discussion

Diabetic foot is one of the chronic complications of diabetic mellitus, with *Staphylococcus aureus* being the most common bacterial infection. With the advent of antibiotics and their frequent use in clinical practice, there is also an increasing amount of antibacterial-resistant bacteria. Severe infections caused by MRSA are an example of this [30]. For diabetic patients with bacterial infections, an ideal drug should have both hypoglycemic and antibacterial effects. As a drug used to treat type 2 diabetes, canagliflozin not only works to reduce blood sugar levels but also has many other functions, including cardiovascular protection, anti-inflammatory effects, and gut microbiota regulation activities. Our study suggest that canagliflozin also had antibacterial activity against MRSA, and its antibacterial activity can be enhanced when combined with penicillin.

**Figure 7:**
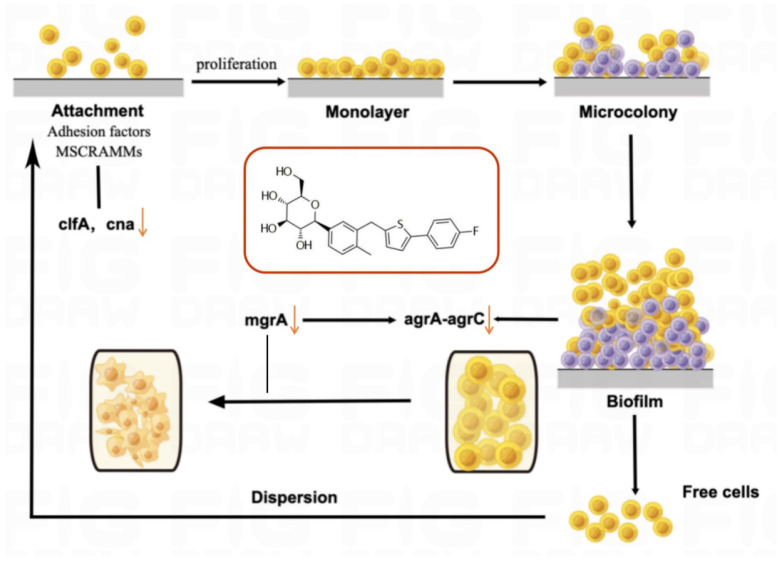
Antibacterial mechanism of canagliflozin. ↓ means downregulation.

The formation of biofilms is one of the crucial reasons why drug-resistant bacterial infections are difficult to eradicate and treat [31,33]. The dynamic process of biofilm formation includes four stages: adhesion, aggregation, maturation, and detachment. In low-density bacterial cell environments, biofilm growth relies on MSCRAMMs to adhere to abiotic substrates [31,34]. We found that canagliflozin downregulated the expression of MSCRAMMs such as clfA and cna, thus affecting the adhesion in the initial stage of biofilm formation. Subsequently, MRSA further adheres and aggregates through polysaccharide intercellular adhesin (PIA) until the biofilm matures. The main virulence system of *Staphylococcus aureus* is composed of agrA and agrC, called agrA-agrC system. Upon reaching high cell density, the virulence system agrA–agrC and gene sarA activate [31], releasing toxins such as hla and hld. Additionally, mgrA affects the agrA–agrC system and also impacts cell-wall-related genes (pbp4, fmtB). Our results show that canagliflozin downregulates the expression of agrC and hld, inhibiting the virulence of MRSA. Canagliflozin also distorted the bacteria surface morphology according to our FESEM observation. The downregulation of mgrA suggested that canagliflozin may affect the bacterial cell wall, which is consistent with the FESEM results. After maturation of the biofilm, some bacterial cells will escape to a new location to grow and repeat the first stage of adhesion. In conclusion, our results suggest that canagliflozin inhibited biofilm growth by affecting related genes, in turn suppressing bacterial proliferation, which coincided with the results of the crystal violet staining experiment. Notably, the expression of mecA, a gene related to methicillin resistance [30], was also downregulated, suggesting that canagliflozin may impact the drug resistance of MRSA. Molecular docking was performed between the proteins corresponding to these genes and canagliflozin. Among them, sarA, crtM, and fnbA were bound well with canagliflozin. Although the SDS-PAGE results show that canagliflozin does not affect the total bacterial protein amount, canagliflozin might affect proteins related to virulence or biofilm formation (Figure 7).

It is important to recognize that glucose and lactic acid were also affected by increasing drug concentrations, though this may be partly due to a decrease in the overall number of bacteria. However, in the ATP test, the bacterial protein extracted from the drug-treated group and the control group was adjusted to the same concentration. The ATP in the drug-treated group was also reduced. This result suggests that canagliflozin can affect bacterial metabolism.

Overall, we discovered that canagliflozin exhibited antibacterial activity against MRSA and primarily inhibited bacterial growth by affecting biofilm formation. Although the IC50 value of canagliflozin alone against MRSA is relatively high and not suitable for oral antibacterial therapy, it could be considered for topical application [35] in the treatment of DFI. The use of canagliflozin could prevent the bacteria from developing antibiotic resistance. Furthermore, canagliflozin and penicillin had strong additive antibacterial activity. Subsequent studies could consider chemical modifications or synthetic methods [36] to improve the antibacterial effects of canagliflozin. Canagliflozin holds potential research value in its antibacterial activity but requires further in vivo investigation into its exact molecular mechanisms and potential applications in the future.

## 4. Materials and Methods

### 4.1. Materials

Canagliflozin was purchased from Hanxiang Biotechnology Co., Ltd., Shanghai, China. Penicillin V potassium was obtained from Sangon Biotech Co., Ltd., Shanghai, China. Doxycycline was obtained from MedChemExpress (https://www.medchemexpress.cn (accessed on 20 May 2023)). Nutrient broth medium powder (HB0108-4) and brain–heart extract liquid medium (HB8297-4) were obtained from Hope Bio-Technology Co., Ltd., Qingdao, China. Agar (AB0016H) was purchased from Sangon Biotech Co., Ltd., Shanghai, China. MRSA (ATCC43300) and MSSA (ATCC 6538) strains were obtained from Guangdong Microbial Culture Collection Center (GDMCC). The bacterial protein extraction kit (BC3750-50T) was obtained from Solarbio, Beijing, China. The PAGE Gel Fast Preparation Kit (PG113) was purchased from Epizyme Biomedical Technology Co., Shanghai, China. Brilliant Blue R250 (ST1123-25g) was purchased from Beyotime, Shanghai, China. The lactic acid detection kit was obtained from Jiancheng Bioengineering Institute, Nanjing, China. The glucose detection kit was purchased from Biosino Bio-Technology and Science Incorporation, Beijing, China. The ATP detection kit was purchased from Beyotime, Shanghai, China.

### 4.2. Determination of Half Maximal Inhibitory Concentrations (IC50)

#### 4.2.1. Canagliflozin (CAN)

IC50 is the drug concentration when the antibacterial rate is 50%. According to the relevant standards of the Clinical and Laboratory Standards Institute (CLSI), the IC50 values of different drugs were detected using the microbroth dilution method. A colony was planted in a liquid medium of about 8 mL, and after shaking the bacteria for about 4 h, the absorbance of the bacterial suspension was adjusted to OD_620nm_ = 0.5. Then it was diluted about 400 times in nutrient broth to reach a final bacterial cell concentration of 5×10^5^ CFU/mL (colony-forming units/mL). CAN dissolved in dimethyl sulfoxide (DMSO) was added to the bacterial suspension to achieve final concentrations of 10, 20, 30, 40, 50, 100, and 200 μM. The 200 μL configured sample was then transferred to a 96-well microtiter plate for three repetitions of each concentration. The samples were cultured at 37 °C for 24 h. Considering the rapid growth of MRSA and the instability of CAN, the OD_620nm_ values were determined to be measured with a microplate reader (Epoch; Biotake, Beijing, China) every 2 h for 12 h and at 24 h.

#### 4.2.2. Penicillin (PNC)

MRSA pretreatment was the same as above. Penicillin V potassium freshly dissolved in water was added to the bacterial suspension to achieve the final concentrations of 2.5, 12.5, 25, 37.5, 62.5, and 125 μM (i.e., 1, 5, 10, 15, 20, 25, and 50 μg/mL). The 200 μL configured sample was then transferred to a 96-well microtiter plate for three repetitions of each concentration. The samples were cultured at 37 °C for 24 h, and the OD_620nm_ values were determined to be measured with a microplate reader every 2 h for 12 h and at 24 h.

#### 4.2.3. Doxycycline (DOX) 

MRSA pretreatment was the same as above. Doxycycline dissolved in DMSO was added to the bacterial suspension to achieve final concentration of 0.002, 0.02, 0.1, 0.2, 2, and 10 μM. The 200 μL configured sample was then transferred to a 96-well microtiter plate for three repetitions of each concentration. The samples were cultured at 37 °C for 24 h, and the OD_620nm_ values were measured using a microplate reader every 2 h for 12 h and at 24 h.

### 4.3. Growth Curve and Fractional Inhibitory Concentration Index (FICI)

The combined effect of drugs can be directly reflected by the growth curve and FICI. The strain was grown to OD_620nm_ = 0.5 in a nutrient medium and then diluted about 200 times in that nutrient medium. CAN, PNC, and CAN + PNC were added to the bacterial suspension separately to reach the final concentration (IC50), and at the same time, a nutrient broth containing 0.1% DMSO and 0.1% H_2_O served as a blank group. The CAN group alone needed to supplement 0.1% H_2_O and the PNC group alone needed 0.1% DMSO. Each group had three replicates. The samples were cultured at 37 °C and OD_620nm_ values were measured every two hours for 12 h. This series of growth curves was used to evaluate the combined effect of CAN and PNC. At the same time, CAN, DOX, and CAN + DOX were added to the bacterial suspension to achieve the final concentration (IC50), and the nutrient broth containing 0.1% DMSO was used as the blank group. The remaining steps were the same as above. To calculate the FICI between PNC and CAN, one group was PNC (different concentrations, 12.5, 25, 37.5, 42.5, 50, 62.5, 125 μM) with CAN (IC50 concentration), while the other group was CAN (different concentrations, 30, 40, 50, 60, 70, 80, 90, 100 μM) with PNC (IC50 concentration). The procedure of the FICI between DOX and CAN was the same as above. FICI = MIC_A(combination)_/MIC_A_ + MIC_B(combination)_/MIC_B_. The FICI was interpreted as follows: FICI ≤ 0.5 denotes “synergy”; FICI > 0.5 to ≤ 1 denotes “additivity”; FICI > 1 to ≤4 denotes “no interaction”; FICI > 4 denotes “antagonism”.

### 4.4. Biofilm Growth

In order to explore the antibacterial mechanism of CAN, the formation of bacterial biofilm was measured. MRSA pretreatment was the same as above. Then, 1 mL bacterial suspension was added to each well of a 12-well plate, and CAN was added to reach concentrations of 25, 50, and 100 μM, with three repeats for each concentration. After incubation at 37 °C for 12 h, the bacterial solution was slowly sucked out, and then each well was gently washed with PBS twice, and the biofilms were fixed with paraformaldehyde for 30 min and dried at 60 °C. The biofilms were stained with 0.1% crystal violet dye for 15 min. After the crystal violet was sucked out, the biofilms were washed with PBS and dried again. We added 95% ethanol into the hole and gently shook the plate to make the adsorbed crystal violet completely fall off. Finally, the absorbance was measured at OD_570nm_.

### 4.5. SDS-Polyacrylamide Gel Electrophoresis (SDS-PAGE)

SDS-PAGE was used to analyze the effects of CAN on MRSA bacterial protein levels. MRSA pretreatment was the same as above. CAN was added to the bacterial suspension to reach the final concentrations of 25 and 50 μM. The configured samples were transferred to a 6-well plate at 37 °C with 2 mL per well and cultured for 12 h. Then, the samples were collected and centrifuged at 10,000× *g* at 4 °C for 5 min. The supernatants were discarded, and the bacteria pellets were washed twice with PBS. The bacterial lysates were added according to the respective OD values, i.e., (OD − OD_0h_) × 200 μL/(OD_ctrl_ − OD_0h_). After adding the bacterial lysate, ultrasonic cracking was performed for 10 min per sample, followed by centrifugation at 8000× *g* at 4 °C for 10 min. Next, the supernatants were collected, and a loading buffer of 20 μL was added to the 80 μL supernatant. The loading buffer contained 100 mmol/L Tris-HCl pH 6.8, 10% sodium dodecyl sulfate (SDS), 0.5% bromophenol blue, 50% glycerine, and 200 mmol/L dithiothreitol (DTT). The prepared samples were then boiled at 100 °C for 10 min, cooled on ice, and analyzed using SDS-PAGE. After electrophoresis, the protein bands were stained with Coomassie Brilliant Blue R-250 and then decolorized to obtain the separated protein bands. The results of SDS-PAGE were photographed with a Gel Imager System (Thermo Fisher, Shanghai, China).

### 4.6. ATP Detection

The pretreatment was the same as that of SDS-PAGE. The collected supernatants were prepared according to the instructions of the ATP detection kit. Amounts of 100 μL ATP working liquid and 20 μL sample were added to each hole of the 96-well plate, and the counts per second (Cps) values were determined using a chemiluminescence analyzer.

### 4.7. Glucose Detection

As the broth medium did not contain glucose, MRSA was cultured in a brain–heart extract liquid medium instead. The rest of the pretreatment was the same as above. An amount of 1 mL of bacterial suspension was added to each well of the 12-well plate, and then CAN was added to reach concentrations of 25, 50, and 100 μM, with three repeats for each concentration. As MRSA thrived, glucose in the medium was consumed after 12 h of culture, so the glucose content was detected at about 4 h of culture. After incubation at 37 °C for 4 h, the bacterial solution was collected and centrifuged at 8000× *g* for 10 min. The supernatants (i.e., culture medium) were collected, and tests were carried out according to the instructions of the glucose kit. Amounts of 200 μL glucose detection reagent and 2µL sample were added to each well of the 96-well plate. Brain–heart extract liquid medium was the control group. All samples were held at 37 °C for 10 min, and then their OD_505nm_ values were measured.

### 4.8. Lactic Acid Detection

MRSA was cultured in a brain–heart extract liquid medium. The rest of the pretreatment was the same as above. An amount of 1 mL bacterial suspension was added to each well of the 12-well plate, and then CAN was added to reach concentrations of 25, 50, and 100 μM, with three repeats for each concentration. After incubation at 37 °C for 4 h, the bacterial solution was collected and centrifuged at 8000× *g* for 10 min. The supernatants (i.e., culture medium) were collected and tests were carried out according to the instructions of the lactic acid kit. The sample, enzyme working fluid, and chromogenic agent were mixed at the ratio of 1:50:10. The brain–heart extract liquid medium was used as the control. After samples were placed for 10 min in a water bath at 37 °C, the termination solution was added (termination solution: chromogenic agent = 10:1), and then their OD_530nm_ values were measured.

### 4.9. Field-Emission Scanning Electron Microscopy (FESEM)

FESEM was performed as described by Wang [28], with some modifications. The bacterial suspension (at least 10 mL) was centrifuged at 8000× *g* for 10 min. Then the supernatant was discarded to obtain the bacterial cells and 1 mL of 2.5% glutaraldehyde was added. After resting overnight, the bacterial cells were cleaned three times with PBS for 30 min each time, and then gradient dehydration was carried out with ethanol (the concentrations were 30%, 50%, 70%, 85%, and 90%, for 15 min each time and 100% dehydration twice). We added 100% tert-butanol to replace ethanol and repeated the procedure 2–3 times, each time at 4 °C for 30 min. Finally, tert-butanol was added, and the samples were frozen at −80 °C for 12 h and then freeze-dried for 12 h. Specimens were attached to metallic stubs using carbon stickers and sputter-coated with gold for 30 s. Then, imaging was performed at different magnifications of 5.00 K SE (L) or 10.00 K SE (L) with FESEM (SU8010, HITACHI, Japan).

### 4.10. RNA Extraction and Real-Time PCR

MRSA pretreatment was the same as above. CAN was added to the bacterial suspension to the final concentration of 50 μM as the drug-treated group. The expression of virulent genes in the control and CAN-treated MRSA strain was evaluated using quantitative real-time reverse transcription polymerase chain reaction (qPCR) following the method of Simran Sinsinwar [29], with some modifications. After bacterial culture for 12 h, the bacterial suspensions were collected and centrifuged at 8000× *g* for 10 min. The supernatant was discarded, and the precipitated cells were washed with DEPC-treated water. RNA samples were extracted and isolated with Trizol reagent and chloroform. Isopropyl alcohol was then added and centrifuged at 10,000× *g* for 15 min to allow bacterial RNA to precipitate. After discarding the supernatant, we added ethanol, and then centrifuged the sample to remove the ethanol. Finally, 20 μL of DEPC-treated water was added to obtain bacterial RNA. The concentration of RNA was measured using a Nanodrop (Thermo Fisher, Shanghai, China). The obtained RNA sample (adjusted to 500 ng) was converted to cDNA by using the PrimeScript RT reagent kit (TransGen Biotech, Beijing, China). The cDNA was amplified in qPCR instruments (qTOWER2.2, analytikjena, Jena, Germany) using SYBR Green (TransGen Biotech, Beijing, China). qPCR was used to study the transcriptional expression level of selected genes such as clfA, cna, agrC, mgrA, hld, mecA, and 16S rRNA (internal reference). All the primers used in the present study and program settings of qPCR are listed in Supplementary Tables S1 and S2. The quantified studied parameters were normalized with housekeeping gene 16S rRNA and expressed as the relative fold change as compared with the control.

### 4.11. Molecular Docking

To analyze the binding affinities and modes of interaction between CAN and targets of MRSA, AutoDockTools-1.5.7, a protein–ligand docking program, was employed. The molecular structures of CAN were retrieved from the PubChem Compound (https://pubchem.ncbi.nlm.nih.gov/) (accessed on 20 May 2023). The 3D coordinates of related proteins were downloaded from the PDB (http://www.rcsb.org/pdb/home/home.do, accessed on 20 May 2023) [30]. For docking analysis, all protein and molecular files were converted into PDBQT format with all water molecules excluded and polar hydrogen atoms were added. The grid box was centered to cover the domain of each protein and to accommodate free molecular movement. The formats of the results were converted into PDB format using Open Bable GUI (software that converts file formats, v. 2.3.1). The results were analyzed and plotted using PyMol-2.2.0 software.

### 4.12. Statistical Analysis

GraphPad Prism 9.0 software was used for statistical analysis. Data were expressed as mean ± standard deviation (S.D.). Differences with statistical significance between groups were calculated via ANOVA followed by Tukey’s post hoc test. *p* < 0.05 was considered statistically significant.

## Data Availability

The data presented in this study are available in the article.

## Supplementary Materials

The following supporting information can be downloaded at: https://www.mdpi.com/article/10.3390/molecules28155668/s1: Figure S1: (A) MSSA growth curves at different concentrations of PNC, (B) MSSA growth curves at different concentrations of DOX; Figure S2: SDS-PAGE analysis of intracellular soluble proteins of MRSA treated with CAN of 25 μM and 50 μM for 12 h; Figure S3: Molecular docking between CAN and bacterial protein active sites; Table S1: List of primers used for qPCR analysis; Table S2: Thermal cyclic conditions used for qPCR analysis.

## Sample Availability

All samples are available in a repository or online in accordance with funder data retention policies.
