# Supplementary material for: Antibacterial Activity and Mechanism of Canagliflozin against Methicillin-Resistant Staphylococcus aureus
Source: Molecules. 2023 Jul 26;28(15):5668. doi: 10.3390/molecules28155668 (PMC10420104; doi:10.3390/molecules28155668)
Supplement: Supplementary file 1 [file molecules-28-05668-s001.zip › molecules-2488481-supplementary.pdf]

Table S1. List of primers used for qPCR analysis.

Primer	(5'to3')	Base number
16S rRNA-F	CATGCTGATCTACGATTACT	20
16S rRNA-R	CCATAAAGTTGTTCTCAGTT	20
mecA-F	GTTAGATTGGGATCATAGCGTCATT	25
mecA-R	TGCCTAATCTCATATGTGTTCCTGTAT	27
mgrA-F	GGGATGAATCTCCTGTAAACG	21
mgrA-R	TTGATCGACTTCGGAACG	18
fnbA-F	ATCAGCAGATGTAGCGGAAG	20
fnbA-R	TTTAGTACCGCTCGTTGTCC	20
cna-F	AAAGCGTTGCCTAGTGGAGA	20
cna-R	AGTGCCTTCCCAAACCTTT	19
crtM-F	ATCCAGAACCACCCGTTTTT	20
crtM-R	GCGATGAAGGTATTGGCATT	20
clfA-F	ATTGGCGTGGCTTCAGTGCT	20
clfA-R	CGTTTCTTCCGTAGTTGCATTTG	23
hld-F	TAATTAAGGAAGGAGTGATTTCAATG	26
hld-R	TTTTTAGTGAATTTGTTCACTGTGTC	26
agrC-F	CATTCGCGTTGCATTTATTG	20
agrC-R	CCTAAACCACGACCTTCACC	20
sarA-F	CAAACAACCACAAGTTGTTAAAGC	24
sarA-R	TGTTTGCTTCAGTGATTCGTTT	22

Table S2. Thermal cyclic conditions used for qPCR analysis

**Initial denaturation**	**95°C for 1 min**	
**Denaturation**	95°C for 15 sec	**40 cycles**
**Annealing**	60°C for 45 sec	
**Extension**	72°C for 6 sec	

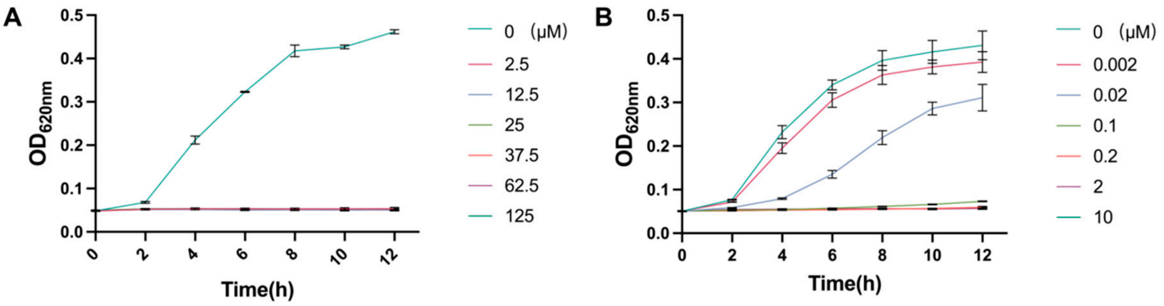

Figure S1. (A)MSSA growth curves at different concentrations of PNC. (B)MSSA growth curves at different concentrations of DOX.

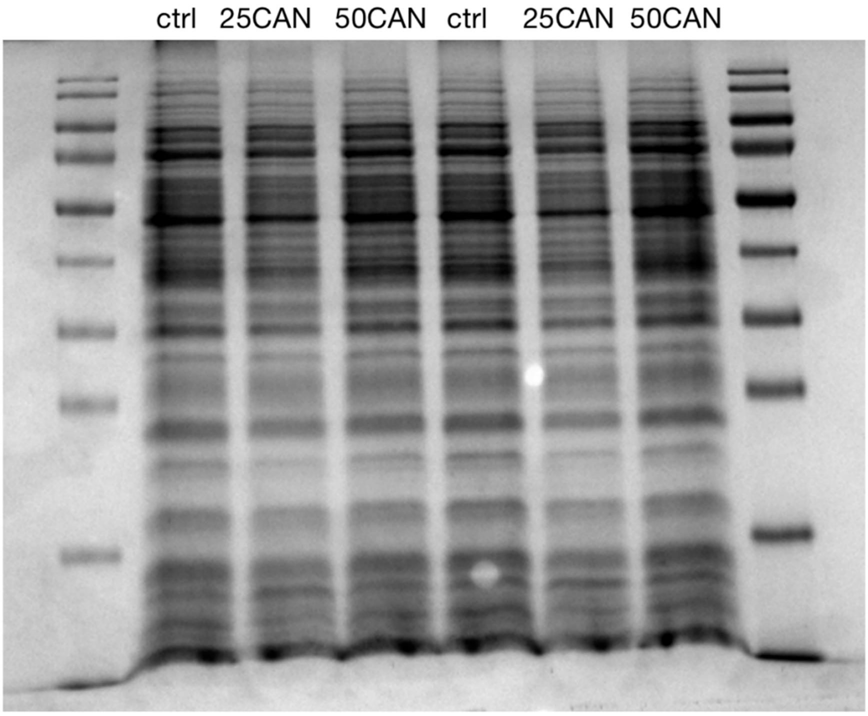

Figure S2. SDS-PAGE analysis of intracellular soluble proteins of MRSA treated with CAN of 25μM and 50μM for 12 hours.

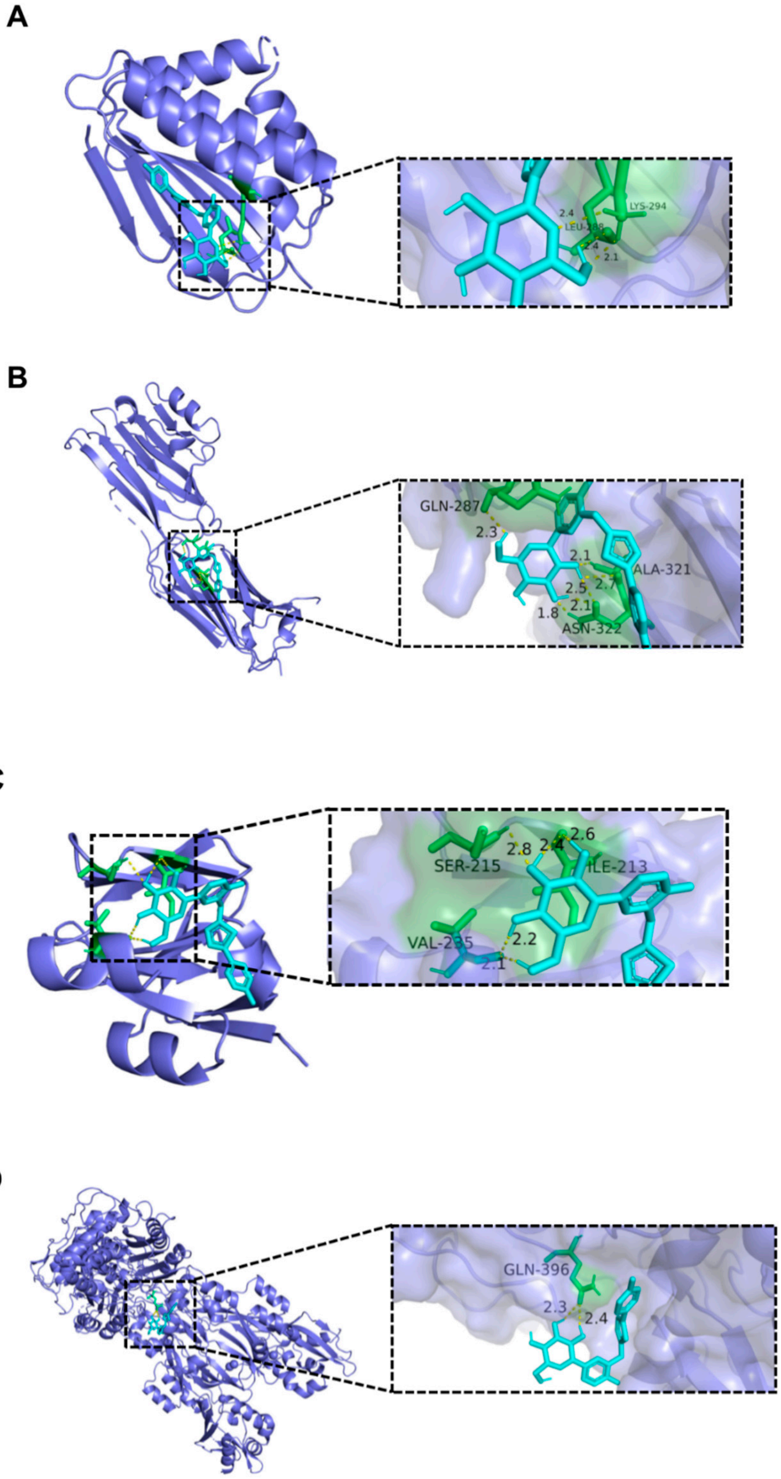

Figure S3. Molecular docking between CAN and bacterial protein active sites. (A) The binding poses and interactions of CAN with agrC, binding energy: -5.55 kcal/mol.
(B) The binding poses and interactions of CAN with cna, binding energy: -5.04 kcal/mol. (C) The binding poses and interactions of CAN with agrA, binding energy: -4.85 kcal/mol. (C) The binding poses and interactions of CAN with PBP2a, binding energy: -3.8 kcal/mol.
